# Social Frailty and Incident Dementia Risk among Community-Dwelling Older Adults

**DOI:** 10.1093/geroni/igaf122.1627

**Published:** 2025-12-31

**Authors:** Claire Wang, Sofia Liu, Anna Beeber, Katherine Ornstein, Rendong He, Junxin Li

**Affiliations:** Johns Hopkins University, Baltimore, Maryland, United States; Johns Hopkins University, Baltimore, Maryland, United States; Johns Hopkins University, Baltimore, Maryland, United States; Johns Hopkins University, Baltimore, Maryland, United States; Johns Hopkins University, Baltimore, Maryland, United States; Johns Hopkins University, Baltimore, Maryland, United States

## Abstract

While social engagement is increasingly recognized as a key determinant of cognitive aging, the broader construct of social frailty—which includes dimensions such as social support, engagement, and resource access—remains understudied as a predictor of dementia risk among community-dwelling older adults. This study draws on data from the National Health and Aging Trends Study (NHATS) collected at Round 12 (baseline) and a one-year follow-up to examine whether baseline social frailty predicts incident dementia. We adapted Makizako’s (2018) social frailty framework by selecting items from its five original domains: (1) being alone; (2) reduced outings; (3) infrequent visits with friends/family; (4) a diminished sense of usefulness; and (5) a lack of daily conversation. Participants were classified as robust (score=0), socially pre-frail (score=1), or socially frail (score=2-5). Among 4,804 participants without dementia at baseline, 5.71% progressed to incident dementia within one year. In the unadjusted logistic analysis, compared to the robust group, social pre-frailty was significantly associated with 38% higher odds of dementia, whereas social frailty had a near 151% higher odds of dementia. In the fully adjusted model, which accounted for demographic and baseline health conditions, this association was attenuated but remained significant for the frail group (OR = 1.41, 95% CI: 1.02–1.93). These findings underscore the importance of social frailty as a predictor of dementia risk. Future research should extend the follow-up period to better understand the mechanisms linking social frailty to cognitive decline and explore innovative interventions that may mitigate dementia risk among community-dwelling older adults.

